# *Amorphophallus konjac*: traditional uses, bioactive potential, and emerging health applications

**DOI:** 10.3389/fpls.2025.1530814

**Published:** 2025-02-21

**Authors:** Archana Jain, Surendra Sarsaiya, Qihai Gong, Qin Wu, Jingshan Shi

**Affiliations:** ^1^ Key Laboratory of Basic Pharmacology and Joint International Research Laboratory of Ethnomedicine of Ministry of Education, Zunyi Medical University, Zunyi, China; ^2^ Bioresource Institute for Healthy Utilization, Zunyi Medical University, Zunyi, China

**Keywords:** *Amorphophallus konjac*, konjac glucomannan, dietary fiber, health benefits, antiinflammatory, multi-omics

## Abstract

*Amorphophallus konjac* is a perennial plant native to Southeast Asia, renowned for its edible corms and rich nutritional value. The bioactive component, konjac glucomannan (KGM), has garnered significant attention due to its broad applications. This review aims to provide a comprehensive overview of the traditional uses, chemical and physical properties, and modern health applications of KGM. It highlights cutting-edge research, discusses challenges and limitations, and identifies future directions for advancing the utility of KGM in health and nutrition. KGM demonstrates remarkable health benefits, including improving metabolic health through weight management, blood glucose stabilization, and lipid profile enhancement. It also plays a vital role in gut health. Emerging evidence highlights its anti-inflammatory and immune-regulatory effects, with applications in managing inflammatory bowel disease, hyperthyroidism, and colorectal cancer (CRC). Recent advancements in multi-omics analyses and high-throughput screening (HTS) approaches have improved KGM extraction, characterization, and evaluation. However, potential side effects such as gastrointestinal discomfort and allergenicity, along with challenges in maintaining purity and molecular consistency, require careful consideration. KGM is a versatile dietary fiber with extensive applications in functional foods, nutraceuticals, and therapeutic interventions. Future research should focus on enhancing KGM's bioavailability, developing targeted delivery systems, and formulating novel applications.

## 1 Introduction

*Amorphophallus konjac*, commonly known for its edible corm and the production of KGM, has a rich cultural and medical history, particularly in East Asia (Pedrosa L de and Fabi, 2024). Traditionally, KGM has been utilized in Chinese medicine for over 2000 years, serving various health purposes such as detoxification, tumor suppression, and treatment of respiratory and skin disorders (Islam et al., 2023). This historical context underscores the significance of konjac in both dietary and medicinal practices, highlighting its role as a staple food source in countries like China and Japan (Ye et al., 2021). The chemical and physical properties of KGM contribute to its health benefits. KGM is a soluble dietary fiber that exhibits unique gel-forming capabilities, which are essential for its applications in food and health products (Sun et al., 2023). Modern research has revealed that KGM can significantly lower plasma cholesterol levels, improve carbohydrate metabolism, and enhance bowel movement, thereby promoting gut health (Du et al., 2021). As a prebiotic, KGM supports the regulation of gut microbiota, which is crucial for maintaining digestive health and overall metabolic function (Xia et al., 2024).

In terms of metabolic health, KGM has shown promise in weight management and blood sugar regulation, making it a valuable component in diabetes management strategies (Fang et al., 2023a). Additionally, its anti-inflammatory properties have been studied for their potential to manage chronic diseases and improve immune function, further expanding its therapeutic applications (Liu et al., 2025). Emerging research is increasingly focused on the diverse biological activities of KGM, exploring its potential in treating lifestyle-related diseases such as obesity and diabetes (Dai et al., 2019). Advanced methodologies, including multi-omics analysis and HTS, are being employed to enhance the extraction, purification, and functional evaluation of KGM, paving the way for innovative health applications (Liu et al., 2023). Emerging research has begun to uncover the broader potential of konjac in addressing metabolic disorders, particularly in combating rising global rates of obesity and type 2 diabetes (Au-Yeung et al., 2018). Its ability to low-density lipoprotein cholesterol (LDL-C) levels and improve blood lipid profiles has positioned it as a promising natural intervention for cardiovascular health. Moreover, the prebiotic properties of glucomannan foster a healthy gut microbiome, offering therapeutic possibilities for managing conditions such as irritable bowel syndrome (IBS) and other digestive disorders (Lasrado and Rai, 2022). This growing body of evidence underscores the need for further research on the mechanisms of action of konjac and its full spectrum of health benefits (Wilianto et al., 2024). As the world increasingly turns to plant-based and natural solutions for health management, *A. konjac* stands out as a traditional remedy with vast modern relevance, offering both preventative and therapeutic potential in the context of contemporary health challenges (Pan et al., 2024). Despite the promising benefits of KGM, challenges remain in standardizing its commercial production and ensuring quality control across different markets. Future research should address these limitations while exploring the full spectrum of KGM's health benefits and applications. This review was aimed to provide a comprehensive overview of the traditional uses, modern health applications, and cutting-edge research surrounding KGM, ultimately contributing to a deeper understanding of its potential in health and nutrition. This review article stands out by offering a holistic view of *A. konjac*, from its traditional uses to its modern health applications, supported by scientific research and innovative approaches like multi-omics analysis and HTS.

## 2 Traditional uses and historical background: an overview of the cultural and medical background of KGM

*A. konjac*, commonly known as konjac, is a perennial plant native to Southeast Asia, particularly valued for its edible corms and traditional applications in food and medicine. This plant has been cultivated for thousands of years, not only as a food source but also for its medicinal properties in traditional Chinese medicine (TCM) (Derosa et al., 2024). The cultivation practices of *A. konjac* involve planting corms, where factors such as corm size and plant density play a crucial role in determining the yield (Xu et al., 2024). Globally, *A. konjac* is produced in significant quantities, with major cultivation occurring in China and Japan, and its production is expanding into other Southeast Asian countries (Luo et al., 2024). The plant prefers warm subtropical to tropical climates, requiring well-drained, nutrient-rich soils that retain moisture without becoming waterlogged (Srzednicki and Borompichaichartkul, 2020). Additionally, moderate shading (50%-70%) is beneficial as it enhances photosynthesis and increases corm weight, making it an essential factor in cultivation (Qin et al., 2019; Nurshanti et al., 2023). The economic importance of *A. konjac* cannot be overstated, as it is in high demand within the food and health industries, primarily due to its glucomannan content, which is valued for its health benefits (Chua et al., 2010). The extraction of KGM from the corms is a key aspect of its commercial processing. This process typically involves washing, slicing, and drying the corms to obtain the soluble fiber used in various applications, including food products and dietary supplements (Kapoor et al., 2024; Widjanarko et al., 2024). The nutritional composition of *A. konjac* corms is noteworthy, as they are rich in glucomannan, starch, and various inorganic elements, contributing to their value as a dietary component (Kulkarni et al., 2024). Despite its benefits, the cultivation of *A. konjac* faces several challenges, including environmental factors, disease management, and the need to optimize growth conditions to enhance yields (Niu et al., 2024; Rahman et al., 2024). These challenges can impact the overall production and availability of *A. konjac* in the global market.

**Figure 1:**
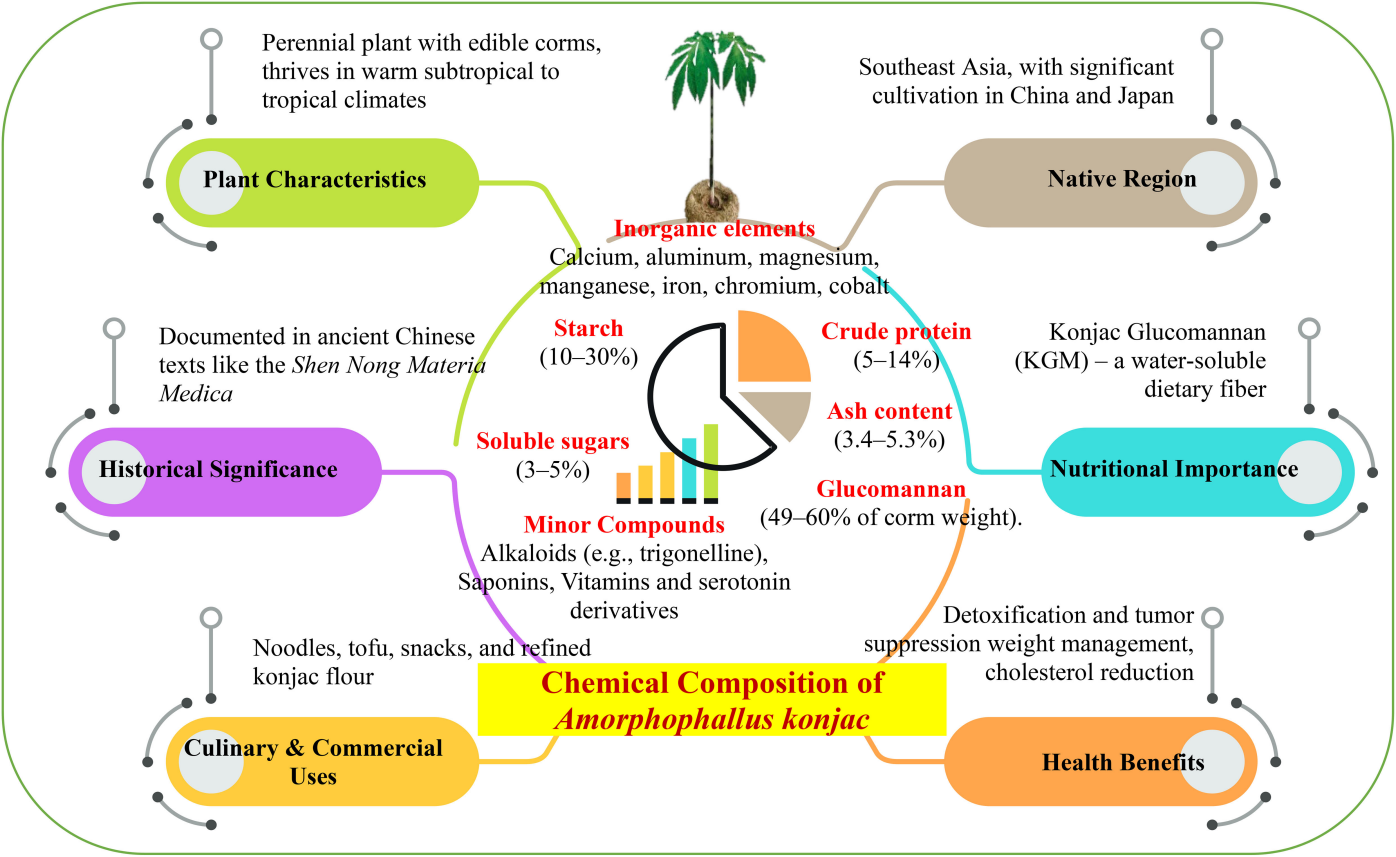
*Amorphophallus konjac*: a plant of culinary, medicinal, and commercial potential.

Its historical significance is underscored by its first documentation in the *'Shen Nong Materia Medica*' during the Western Han Dynasty, which highlights its longstanding role in ancient Chinese medicine (Chua et al., 2010). Over the centuries, konjac has been integrated into various cultural practices, especially in China and Japan, where it has been consumed for its health benefits (Qi et al., 2024). The primary component of konjac is KGM, a water-soluble dietary fiber extracted from its corm. KGM has been utilized in TCM for thousands of years, often for detoxification and tumor suppression (Lin and Rezaei, 2024). The health benefits associated with KGM are well-documented, including its roles in weight management, cholesterol reduction, and digestive health (Devaraj et al., 2019a). Research indicates that KGM can enhance satiety, reduce body weight, and improve metabolic parameters by increasing the transit time of food and prolonging gastric emptying (Shang et al., 2020; Guo et al., 2021). Culinary applications of konjac are also significant, as it is used to create various dishes such as noodles, tofu, and snacks, showcasing its versatility as a food ingredient (Chua et al., 2010; Zhang et al., 2020). The refined konjac flour, which contains a high percentage of KGM, has been introduced into Western markets as a food additive and dietary supplement, reflecting its growing global appeal (Zhu, 2018). Modern research has focused on the extraction, characterization, and health benefits of KGM, expanding its applications in nutrition and medicine (Devaraj et al., 2019a). Studies have demonstrated that KGM possesses pharmacological properties, including anti-obesity and cholesterol-lowering effects, making it a valuable component in health-related products (**Table 1**; **Figure 1**) (Behera and Ray, 2016).

**Table 1:** Overview of Konjac: cultivation, historical significance, and applications.

Aspect	Details	Citations
Plant Origin and Cultivation		
Native Region	Perennial plant native to Southeast Asia, valued for edible corms and medicinal use.	(Derosa et al., 2024)
Cultivation Areas	Primarily cultivated in China, Japan, and other Southeast Asian countries.	(Luo et al., 2024)
Optimal Growth Conditions	Warm subtropical to tropical climates, nutrient-rich soils, and moderate shading.	(Mekkerdchoo et al., 2016; Qin et al., 2019; Nurshanti et al., 2023)
Yield Factors	Corm size and plant density significantly affect yield.	(Xu et al., 2024)
Cultivation Challenges	Environmental factors, disease management, and optimizing growth conditions.	(Niu et al., 2024; Rahman et al., 2024)
Historical Significance		
Ancient Documentation	Mentioned in *Shen Nong Materia Medica* during the Western Han Dynasty.	(Chua et al., 2010)
Cultural Integration	Incorporated into traditional Chinese and Japanese practices for food and health benefits.	(Qi et al., 2024)
Nutritional and Economic Value		
Nutritional Composition	Rich in glucomannan, starch, and inorganic elements.	(Kulkarni et al., 2024)
Economic Demand	High demand in food and health industries due to glucomannan content.	(Chua et al., 2010)
Food Applications	Used in various food products, including noodles, tofu, and snacks.	(Chua et al., 2010; Zhang et al., 2020)
Market Expansion	Refined konjac flour introduced into Western markets as a food additive and dietary supplement.	(Zhu, 2018)
Konjac Glucomannan (KGM)		
Description	Water-soluble dietary fiber with applications in food, medicine, and health products.	(Devaraj et al., 2019b)
Traditional Uses	Utilized in TCM for detoxification and tumor suppression.	(Lin and Rezaei, 2024)
Health Benefits	Weight management, cholesterol reduction, and improved digestive health.	(Shang et al., 2020; Guo et al., 2021)
Pharmacological Properties	Includes anti-obesity and cholesterol-lowering effects.	(Behera and Ray, 2016)
Processing and Applications		
Extraction Process	Involves washing, slicing, and drying corms to obtain soluble fiber.	(Kapoor et al., 2024; Widjanarko et al., 2024)
Research Focus	Modern studies focus on extraction, characterization, and expanded applications.	(Devaraj et al., 2019b)

## 3 Chemical and physical properties of *Amorphophallus konjac*

*A. konjac* is rich in various bioactive compounds, making it both nutritionally and pharmacologically valuable. The predominant compound in these corms is glucomannan, which accounts for approximately 49–60% of the corm weight (Alamgir, 2018). Additionally, corms contain 10–30% starch and 2.6–7% inorganic elements, including key minerals such as calcium, aluminum, magnesium, manganese, iron, chromium, and cobalt (Gómez et al., 2017). These corms also possess crude protein content ranging from 5% to 14%, 3% to 5% soluble sugars, and 3.4–5.3% ash. Furthermore, small quantities of bioactive compounds, such as alkaloids (such as trigonelline) and saponins, are present, particularly near the stem base (Sengupta et al., 2022). In addition to the essential organic and inorganic compounds, *A. konjac* corms are abundant in vitamins and other bioactive components. Corms are notable for their content of some organic compounds, which enhance their nutritional value. Furthermore, fresh corm tissue contains serotonin and its derivatives. These serotonin derivatives are interesting because of their potential therapeutic properties (Shi et al., 2019). The precise chemical composition of mature Amorphophallus corms can vary depending on species, geographic region, and environmental factors during cultivation. Among the nine species of Amorphophallus grown in China, *A. konjac* and *A. albus* are especially important for storing glucomannan as their primary carbohydrate, making them important for both traditional and modern applications (Mekkerdchoo et al., 2016). This variation in composition highlights the importance of understanding the specific growth conditions and species characteristics when utilizing these corms for their health benefits and industrial applications. The study of these variations can lead to optimized cultivation practices, potentially enhancing the yield of desired compounds, such as glucomannan and other bioactive constituents (Gao et al., 2023).

The chemical structure of polysaccharides plays a significant role in determining their functional and nutritional properties as well as their biological activities (Yu et al., 2018). The primary structure of KGM consists of repeating units of D-glucose and D-mannose in a molar ratio of 1:1.6–1.7, connected by β-1,4-glycosidic bonds. Some side chains may branch from the main mannose backbone at the C-3 position or from the sugar unit at the C-6 position through acetyl-group linkages. KGM exists in two native conformations, alpha (amorphous) and beta (crystalline), which affect its physical and functional properties, respectively. The molecular weight of KGM is relatively homogenous and normally distributed, although it can vary depending on factors such as origin, processing methods, and storage conditions. KGM is reported to have an average molecular weight of 5.83 × 10^5^ g/mol, contributing to its unique functional properties in both food and health applications. The unique internal structure of the two-year-old KGM provides insight into the tissue composition of the plant. As the size and quantity of these idioblasts increase toward the center of the corm, the central region contains idioblasts that can reach up to 650 µm in diameter. This branching was relatively minimal, with approximately three branches for each of the 32 sugar units (Felix da Silva et al., 2020). In addition, these calcium oxalate formations are located within KGM-containing idioblasts and in the surrounding parenchyma. The dual presence of KGM and calcium oxalate within the corm tissue highlights the complex structure of *A. konjac* corms. KGM and calcium oxalate are deposited within corms, forming needle-shaped raphide crystals and multi-crystal druses, also known as cluster crystals (Chua et al., 2013). The molecular configuration of KGM includes a mannose-to-glucose ratio of approximately 1.6:1. These acetyl groups are crucial for enhancing the solubility of KGM (Kulkarni et al., 2024). The molecular weight of KGM varies widely, ranging from 200 to 2000 kDa, and is influenced by factors such as the specific cultivar, growing region, and processing and storage techniques used (Liu et al., 2021a). One of KGM’s distinctive properties of KGM is its ability to dissolve in hot and cold water. The solubility of KGM can be further enhanced by applying heat and mechanical agitation, which makes it versatile for various applications across the food and industrial sectors (Shi et al., 2019). KGM solutions may decrease over time, possibly because of bacterial contamination or enzymatic hydrolysis, particularly by β-mannanase. When mild alkali is added to KGM solutions, a thermostable gel is formed, which has applications in food and industrial products (Liu et al., 2021a). This unique combination of structural and physicochemical properties renders KGM a valuable compound for various applications (**Table 2**).

**Table 2:** Chemical composition, structural properties, and applications of konjac glucomannan (KGM).

Aspect	Details	Citations
Main Component	Glucomannan (49–60% of corm weight).	(Alamgir, 2018)
Additional Components		
	Starch (10–30%), inorganic elements (e.g., calcium, magnesium, iron, cobalt).	(Gómez et al., 2017)
	Crude protein (5–14%), soluble sugars (3–5%), and ash (3.4–5.3%).	(Sengupta et al., 2022)
	Bioactive compounds such as alkaloids (e.g., trigonelline), saponins, and serotonin derivatives.	(Shi et al., 2019)
	Vitamins and other bioactive components vary based on growth conditions.	(Gao et al., 2023)
Species Variability		
	*A. konjac* and *A. albus* are key species for glucomannan storage.	(Mekkerdchoo et al., 2016)
	Chemical composition varies with species, region, and environment.	(Gao et al., 2023)
Polysaccharide Structure		
	Repeating units of D-glucose and D-mannose in a 1:1.6–1.7 molar ratio, linked by β-1,4 bonds.	(Yu et al., 2018)
	Side chains branch at C-3 and C-6 positions with acetyl-group linkages.	(Kulkarni et al., 2024)
	Alpha (amorphous) and beta (crystalline) conformations affect physical properties.	(Felix da Silva et al., 2020)
Molecular Properties		
	Molecular weight ranges from 200–2000 kDa; average weight ~5.83 × 10^5^ g/mol.	(Liu et al., 2021b)
	Solubility in hot and cold water, enhanced by heat and mechanical agitation.	(Shi et al., 2019)
	Acetyl groups improve solubility.	(Kulkarni et al., 2024)
	Forms thermostable gel with mild alkali; useful in food and industrial products.	(Liu et al., 2021b)
Corm Tissue Structure		
	Contains KGM and calcium oxalate in needle-shaped raphides and cluster-shaped druses.	(Chua et al., 2013)
	Idioblasts in corms increase in size toward the center; central region ~650 µm diameter.	(Felix da Silva et al., 2020)
Physical Properties		
	KGM solutions degrade over time due to bacterial contamination or enzymatic hydrolysis.	(Shi et al., 2019)
	Unique gelation and functional properties for food, health, and industrial applications.	(Liu et al., 2021b)

## 4 Modern health applications

KGM is a soluble fiber derived from the konjac plant, known for its potential health benefits, particularly in managing metabolic disorders. One of the primary advantages of KGM is its ability to ameliorate conditions such as diabetes and hypercholesteremia, suggesting its role as a functional food in metabolic health (Jian et al., 2024). KGM is recognized as an effective weight loss aid, promoting satiety and reducing overall caloric intake. This is largely due to its capacity to form a gel-like substance in the stomach, which enhances feelings of fullness and helps control hunger (Jin et al., 2024). Consequently, individuals may experience reduced food intake, contributing to weight management efforts. Moreover, KGM supports digestive health by slowing down the digestive process, which can improve bowel movements and overall gut function (Deng et al., 2024). This property not only aids digestion but also plays a role in regulating blood sugar levels by slowing the absorption of glucose in the bloodstream after meals, thereby helping to maintain stable blood sugar levels (Fang et al., 2023a). The health benefits of KGM encompass weight loss support, appetite suppression, improved digestive health, and blood sugar regulation, making it a valuable addition to a balanced diet aimed at enhancing overall well-being (**Table 3**).

**Table 3:** Health benefits and mechanisms of konjac glucomannan (KGM).

Health Aspect	Mechanism of Action	Findings	Advantages	Citations
Metabolic health (e.g., diabetes, weight loss)				
Weight Management	Forms a gel-like substance in the stomach, increasing satiety and reducing caloric intake.	5.5 lbs weight loss in 8 weeks with a low-calorie diet.	Aids adherence to dietary regimens and promotes healthy weight loss.	(Behera and Ray, 2016; Hasbay, 2019; Mah et al., 2022)
		8.5% reduction in body fat among overweight children in 2 months.	Effective in children and adults for weight and fat reduction.	(Ngondi et al., 2005; Behera and Ray, 2016)
		51 ± 16% reduction in excess weight among obese children over 4 months.	Supports pediatric obesity management.	(Behera and Ray, 2016)
Blood Glucose Regulation	Slows glucose absorption, stabilizing blood sugar levels and enhancing insulin sensitivity.	Fasting glucose reduction by 23.2% in type 2 diabetics.	Improves long-term glycemic control (HbA1c).	(Shah et al., 2015b; Behera and Ray, 2016)
		Glucose levels decreased by 55.37% with 1.5 g/kg dose in studies.	Helps manage diabetes and prediabetes.	(Ling et al., 2013)
Lipid Profile Improvement	Binds bile acids, promoting cholesterol excretion, reducing LDL-C and triglycerides.	LDL-C reduction by 5.45%; TC decreased by 3.24%.	Reduces cardiovascular risk factors like LDL-C and TC.	(Reimer et al., 2013; Li et al., 2021)
		More pronounced cholesterol reduction in females (LDL-C 9%, TC -6.1%).	Gender-specific benefits observed in lipid improvements.	(Vuorio et al., 2017; Ding et al., 2020)
		Cholesterol reduced by 7.3% in hypercholesterolemic children.	Safe and effective for managing lipid profiles in children.	(Santos et al., 2011)
Digestive Health	Acts as a soluble fiber, slowing digestion and enhancing gut function.	Promotes regular bowel movements and improved gut microbiota composition.	Supports overall digestive well-being.	(Kapoor et al., 2024)
Childhood Health	Combats obesity and hypercholesterolemia through a low-fat, fiber-rich diet.	Significant weight and cholesterol reductions observed in pediatric studies.	Offers a safe approach to improving metabolic health in children.	(Santos et al., 2011; Behera and Ray, 2016)
Dosage Variability	Dosages ranging from 0.7 g to 15 g/day show varying efficacy based on BMI and health conditions.	Higher dosages yield more pronounced effects on weight, glucose, and cholesterol management.	Customizable dosages for individual health needs.	(Devaraj et al., 2019b; Fang et al., 2023b)
Additional Benefits	Biocompatible, biodegradable polysaccharides support cardiovascular health and metabolic regulation.	Reduced risk factors like cholesterol, blood sugar, and obesity.	Suitable for functional foods and supplements.	(Watanabe et al., 2020; Kapoor et al., 2024)
Gut Health (e.g., prebiotics, microbiota regulation)				
Gut Microbiota Modulation	Enhances growth of beneficial bacteria like Bifidobacterium and Lactobacillus; increases microbiota diversity.	Promotes a balanced gut environment and reduces CRC risk through microbiome modulation.	Supports gut health and reduces risks of colorectal diseases.	(Wu et al., 2011; Gómez et al., 2017; Li et al., 2024)
Bowel Health and Regularity	Adds stool bulk and regulates bowel movements; dose-dependent effects.	3 g/day increases frequency by 3 evacuations/week; 4 g/day increases by 6 evacuations/week.	Relieves constipation, supports regularity, and improves overall bowel health.	(Hayeeawaema et al., 2020)
Prebiotic Properties	Supports beneficial gut bacteria and balances the gut environment.	Alleviates inflammation and promotes a healthy intestinal barrier.	Acts as a natural prebiotic with anti-inflammatory benefits.	(Du et al., 2021)
SCFAs	Enhances SCFA production, maintaining gut barrier integrity and modulating immune responses.	SCFAs reduce inflammation, improve gut health, and lower CRC risk.	Reduces inflammation and strengthens gut barrier.	(Zhang et al., 2023; Jin et al., 2025)
Colorectal Cancer Prevention	Reduces β-glucuronidase activity and secondary bile acids, minimizing genotoxic risks.	High-fiber diets including KGM lower CRC risk and enhance the efficacy of therapies like immunochemotherapy.	Prevents CRC and supports cancer treatments.	(Sawai et al., 2019; Yan et al., 2024)
IBD Symptom Alleviation	Improves stool consistency, fosters beneficial bacteria, and reduces gut inflammation.	Alleviates diarrhea and constipation symptoms in IBD patients.	Promotes intestinal health and symptom relief in IBD.	(Guarino et al., 2020; Du et al., 2021)
Hyperthyroidism Management	Supports nutrient absorption and regulates gastrointestinal symptoms like diarrhea.	Mitigates malabsorption and diarrhea symptoms while managing weight.	Improves gut health and nutritional balance in hyperthyroid patients.	(Kalra et al., 2021; Gan et al., 2024)
DNA Protection	Reduces harmful metabolites like β-glucuronidase and secondary bile acids that lead to DNA damage.	Protects against DNA damage and cancer development in the colon.	May reduce cancer risks and improve cellular health.	(Deng et al., 2024; Yan et al., 2024)
Therapeutic Role in CRC	Enhances the effectiveness of CRC treatments (e.g., polysaccharide K in immunochemotherapy).	Adjuvant therapies improve survival rates and reduce treatment side effects.	Complements CRC treatment, enhancing outcomes and reducing toxicity.	(Sawai et al., 2019)
Anti-inflammatory and immune regulation				
Skin Inflammation Relief	Lowers IL-4/IFN-γ ratio and suppresses hyper-IgE production.	Reduces symptoms of atopic dermatitis, allergic reactions, and eczema.	Effective in managing atopic dermatitis and other skin allergies.	(Devaraj et al., 2019b; Tian et al., 2023; Pan et al., 2024)
Colitis Management	Reduces cytokines (e.g., IL-4, IL-13, TNF-α, IL-1β) and modulates immune responses.	Improves symptoms of OXA by lowering inflammatory markers.	Supports treatment of inflammatory bowel conditions like colitis.	(Handa et al., 2015; Onitake et al., 2015; Fang et al., 2023b)
Gut Inflammation Modulation	Regulates gut inflammation through cytokine reduction and microbiome modulation.	Potential to manage conditions like Irritable Bowel Disease (IBD).	Improves gut health and reduces inflammation in IBD patients.	(Changchien et al., 2021)
Wound Healing	Maintains moisture at the wound site, absorbs exudates, and promotes tissue repair.	Accelerates healing and reduces risk of infection; effective for biocompatible wound care applications.	Provides safe and effective wound management for various skin conditions.	(Alven et al., 2022; Zhou et al., 2022; Borbolla-Jiménez et al., 2023)
Gut Microbiota and Immune Support	Enhances growth of beneficial bacteria (e.g., Bifidobacteria, Lactobacilli) and GALT function.	Improves immune system resilience and enhances GALT activity.	Supports a healthy gut microbiome, which benefits systemic immunity.	(Devaraj et al., 2019b; Ye et al., 2021)
SCFAs Production	Increases SCFAs (e.g., acetate, propionate, butyrate) essential for gut integrity and inflammation control.	Reduces systemic inflammation, strengthens gut barrier, and supports immune tolerance.	Promotes gut health and reduces risks of autoimmune and inflammatory disorders.	(Elshaghabee and Rokana, 2021; Zhang et al., 2022a)
Allergy and Atopic Dermatitis	Suppresses OVA-specific IgE response; reduces hyper-IgE production.	Ameliorates eczema and allergic responses, highlighting its potential for atopic disease management.	Helps manage allergies and atopic conditions like dermatitis and rhinitis.	(Suzuki et al., 2010; Mao et al., 2022; Guerreiro et al., 2023)
Immune Cell Regulation	Supports T-cell and macrophage activity through gut health and beneficial metabolite production.	Enhances immune defense and promotes systemic immune resilience.	Contributes to stronger immunity and balanced immune responses.	(Pan et al., 2024; Srividya et al., 2024)
Anti-inflammatory Mechanisms	Downregulates pro-inflammatory cytokines (e.g., TNF-α, IL-1β) and reduces NK1.1+ T cells.	Reduces systemic and localized inflammation, especially in conditions like colitis.	Effective for immune modulation and controlling chronic inflammatory diseases.	(Handa et al., 2015; Fang et al., 2023b)
Individual Variability	Immune and anti-inflammatory effects depend on individual health conditions and genetic factors.	Benefits vary; consultation with healthcare providers is advised for safe dietary integration in immune-related cases.	Tailored application ensures safety and effectiveness for diverse populations.	(Srividya et al., 2024)

### 4.1 Metabolic health (e.g., diabetes, weight loss)

KGM, derived from the tubers of *A. konjac*, has gained attention for its multifaceted health benefits, particularly in the realm of metabolic health (Behera and Ray, 2016; Devaraj et al., 2019a). Its role in weight management, blood glucose regulation, and lipid profile improvement positions it as a valuable dietary intervention for conditions such as obesity and diabetes (Fang et al., 2023a). One of the primary applications of KGM is its effectiveness in promoting weight loss. Research indicates that incorporating glucomannan into a low-calorie diet significantly enhances weight loss outcomes compared to a diet alone (Jian et al., 2024). In a study involving 30 patients, those who consumed glucomannan alongside a 1,200 kcal diet experienced greater reductions in body weight and fat mass, alongside improved satiety and adherence to dietary regimens (Koncz et al., 2021; Mah et al., 2022). This is particularly relevant given the rising prevalence of obesity in many populations. In an 8-week study, participants who added KGM to a hypocaloric diet lost an average of 5.5 lbs, with a notable reduction in body fat, compared to the diet-only group (Hasbay, 2019; Xu et al., 2023). Regular consumption of KGM has been shown to significantly decrease body fat, particularly in individuals with a higher body mass index (BMI). In a clinical trial involving children, the glucomannan treatment group experienced a notable reduction in mean overweight from 49.5% to 41%, indicating a substantial decrease in body fat over the course of the study (Ngondi et al., 2005). Additionally, another study reported that treated obese patients exhibited a significant decrease in excess weight of 51 ± 16% compared to controls after four months, further supporting the effectiveness of KGM in weight reduction (Behera and Ray, 2016).

In addition to weight loss, KGM has demonstrated positive effects on metabolic health by regulating blood glucose levels. The consumption of glucomannan has been associated with improved insulin sensitivity and lower blood sugar levels, which are crucial for managing diabetes (Shah et al., 2015a). This regulation of blood glucose is complemented by KGM's ability to enhance carbohydrate tolerance, further supporting its role in diabetes management (Devaraj et al., 2019a). Research has shown that KGM has demonstrated significant efficacy in reducing glucose levels by 55.37% when administered at a dose of 1.5 g/kg in both animal models and clinical settings (Ling et al., 2013). Additionally, konjac flour, which contains glucomannan, resulted in a 40.9% reduction in blood sugar levels under similar experimental conditions. In clinical trials involving type 2 diabetic patients, KGM supplementation has been associated with a reduction in fasting glucose levels by 23.2% compared to placebo (Shah et al., 2015a; Behera and Ray, 2016). Dosage differences also play a crucial role in the effectiveness of KGM. Studies have reported a wide range of KGM dosages, from 0.7 g to 15 g per day, which can significantly influence the outcomes related to blood sugar regulation and cholesterol reduction (Devaraj et al., 2019a; Fang et al., 2023a). Higher dosages may yield more pronounced effects, while lower dosages might not be sufficient to achieve significant results. Furthermore, the demographic and health characteristics of study participants can affect KGM's efficacy. Factors such as age, BMI, and pre-existing health conditions can lead to variations in how individuals respond to KGM supplementation (Devaraj et al., 2019a; Colantonio et al., 2020). By forming a gel-like substance in the stomach, KGM not only slows the absorption of sugars but also helps regulate the release of insulin (Xia et al., 2024). Improved insulin sensitivity indicates that cells become more responsive to insulin, facilitating better regulation of blood glucose levels (Kapoor et al., 2024). Additionally, some studies suggest that KGM may help lower HbA1c levels, which are indicators of long-term blood sugar control and are vital for preventing diabetes-related complications. In addition to these direct effects, KGM also supports metabolic health through its impact on weight. Additional weight is a main risk factor for Type 2 diabetes, and the ability of KGM to induce satiety can help with weight management and reduce overall caloric intake. This weight-lowering effect can further enhance insulin sensitivity and glycemic control (Watanabe et al., 2020). Additionally, KGM, a soluble dietary fiber derived from the konjac plant, has demonstrated significant potential in improving cholesterol levels, particularly by lowering LDL-C and triglycerides, which are crucial for cardiovascular health. In a controlled study, subjects taking glucomannan experienced a notable reduction in serum cholesterol by −32.0 mg/dl and LDL-C by −28.7 mg/dl, indicating its effectiveness in managing lipid profiles (Behera and Ray, 2016). The mechanism behind KGM's cholesterol-lowering effects is attributed to its ability to bind bile acids in the intestine, promoting their excretion and subsequently leading to a reduction in cholesterol levels as the body utilizes cholesterol to replenish bile acids (Kapoor et al., 2024). Meta-analyses of KGM studies suggest overall benefits in lowering cholesterol and blood glucose, but they also highlight the need for standardized methodologies to improve reliability and comparability of results (Ho et al., 2017). Clinical trials have reported significant reductions in fasting glucose and cholesterol levels with KGM supplementation, yet these results can vary based on trial conditions (Chen et al., 2003; Xia et al., 2024).

**Figure 2:**
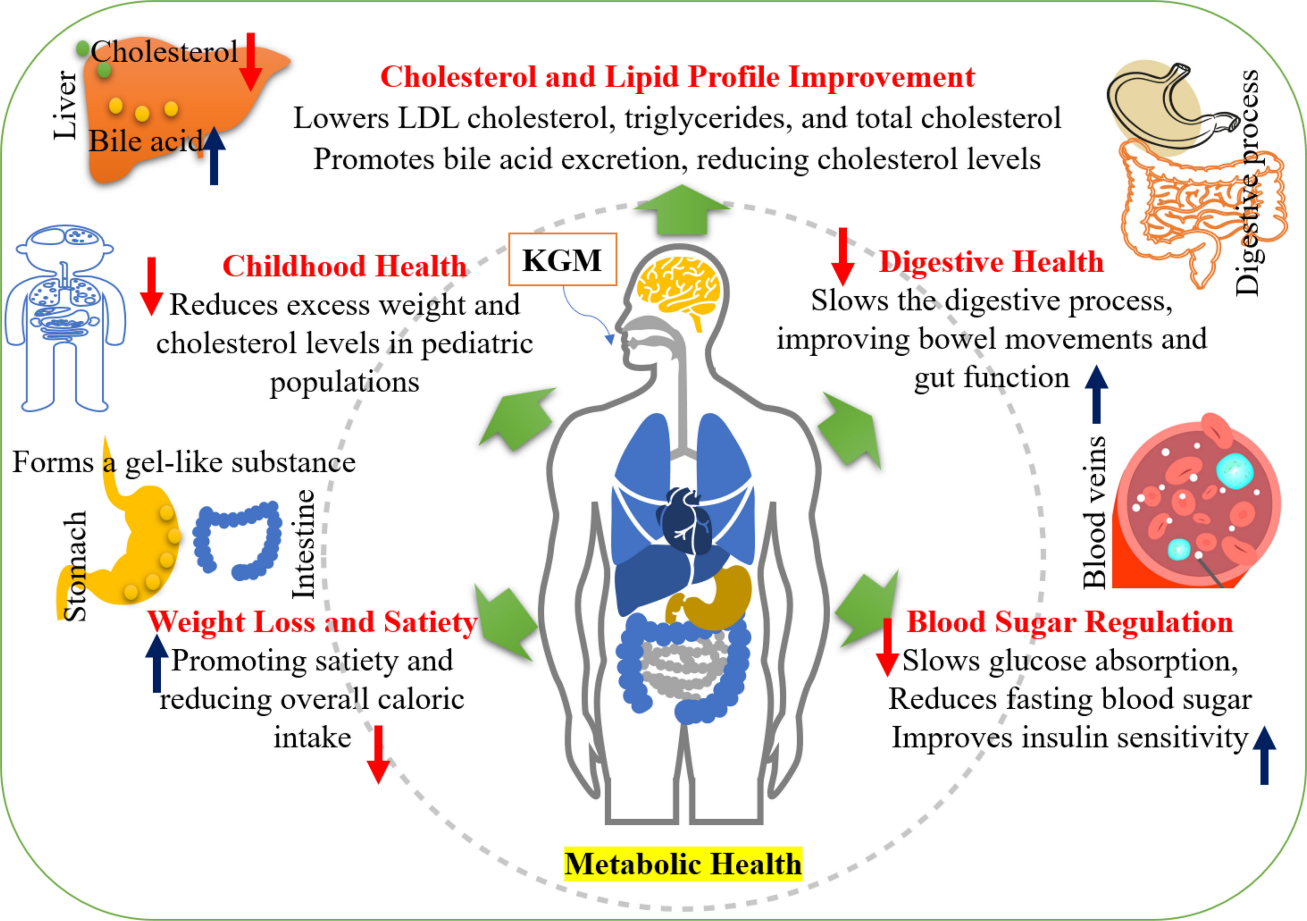
Metabolic health benefits of konjac glucomannan (KGM).

Moreover, KGM contributes to the improvement of lipid profiles, particularly in lowering total serum cholesterol and enhancing overall lipid status. The same study that highlighted weight loss also reported significant improvements in lipid parameters for participants consuming glucomannan, indicating its potential for cholesterol management (Zhu et al., 2024). This is particularly important as elevated cholesterol levels are a major risk factor for cardiovascular diseases. The health benefits of KGM extend beyond individual metrics; its soluble fiber content aids in the maintenance of normal blood cholesterol levels, making it a promising candidate for dietary interventions aimed at improving metabolic health (Kapoor et al., 2024). In a study involving 110 elderly individuals with hyperlipidemia, those who consumed refined konjac meal experienced notable decreases in triglyceride (TG), total cholesterol (TC), and LDL-C levels, along with increases in HDL-C and apoprotein (AI) levels. In contrast, the control group, which did not consume KGM, showed no changes in blood lipid and cholesterol (Mah et al., 2022). In a study assessing the efficacy of Minolest, which contains KGM, participants experienced a significant decrease in TC by 3.24%. Additionally, the study highlighted a more pronounced reduction in LDL-C, which decreased by 5.45% (Reimer et al., 2013; Li et al., 2021). These findings suggest that KGM supplementation can be beneficial for individuals with mild hypercholesterolemia, particularly those at a lower risk for coronary artery disease (Koyama, 2017). KGM has been shown to be effective in managing childhood obesity, particularly in reducing excess weight among obese children. In a clinical study involving 23 obese children aged between 5.2 and 15.8 years, those treated with highly purified glucomannan fibers experienced a significant reduction in excess weight, averaging 51 ± 16% compared to controls. This treatment involved administering 2-3 capsules of glucomannan twice daily, alongside a balanced diet, which ensured adequate caloric intake for all participants (Behera and Ray, 2016). Moreover, another study indicated that children under glucomannan treatment demonstrated a decrease in mean overweight from 49.5% to 41% over a two-month period, highlighting its effectiveness in weight management (Behera and Ray, 2016). In hypercholesterolemic children, the incorporation of KGM into a low-fat, fiber-rich diet has demonstrated significant efficacy in reducing cholesterol levels. Specifically, the treatment resulted in a 5.1% reduction in TC levels and a 7.3% decrease in LDL-C levels (Santos et al., 2011). These findings highlight the potential of KGM as a beneficial dietary intervention for managing cholesterol levels in this population. Moreover, gender differences were observed in the effectiveness of KGM treatment. In females, the reduction in TC was reported at 6.1%, while the decrease in LDL-C was even more pronounced at 9% (Vuorio et al., 2017; Ding et al., 2020). The ability of KGM to act as a natural polysaccharide with excellent biocompatibility and biodegradability further enhances its appeal for use in functional foods and supplements (Chandarana et al., 2024). KGM serves as a powerful tool in modern health applications, particularly for metabolic health. Its efficacy in weight loss, blood glucose regulation, and lipid profile improvement underscores its potential as a dietary intervention for managing obesity and diabetes, making it a significant focus for future research and public health initiatives (**Figure 2**) (Liu et al., 2025).

### 4.2 Gut health (e.g., prebiotics, microbiota regulation)

KGM is a soluble fiber that has garnered attention for its potential to modulate the gut microbiome, which may play a significant role in CRC prevention. One of the primary mechanisms by which KGM exerts its effects is through the enhancement of beneficial gut bacteria, particularly the Bifidobacterium genus and the Lactobacillus–Enterococcus group (Li et al., 2024). These populations significantly increase following the fermentation of KGM, suggesting a favorable modulation of gut microbiota that could contribute to protective effects against CRC (Gómez et al., 2017; Wu et al., 2011). Data from studies indicate that a daily intake of 3 grams of KGM results in an increase of 0.5 times per day and 3 additional evacuations per week compared to baseline values. When the dosage is increased to 4 grams per day, the effect is even more pronounced, with an increase of 0.9 times per day and 6 more evacuations per week. This demonstrates a clear dose-response relationship, where higher doses of KGM correlate with greater improvements in stool bulk and frequency of bowel movements (Hayeeawaema et al., 2020). KGM, a soluble dietary fiber, forms a gel-like substance when it enters the digestive tract. This gel can improve stool consistency, which is particularly beneficial for individuals with digestive disorders such as irritable bowel disease (IBD). KGM's ability to regulate stool consistency may help alleviate symptoms associated with IBD, such as diarrhea and constipation, thus contributing to better overall bowel health and comfort (Guarino et al., 2020).

KGM also acts as a prebiotic that fosters the growth of beneficial gut bacteria. The healthy gut microbiome plays a critical role in maintaining intestinal health and reducing inflammation. By supporting the proliferation of beneficial bacteria, KGM may contribute to a balanced gut environment, which is essential for managing IBD and promoting overall gut health (Du et al., 2021). Maintaining a healthy weight can be a challenge for patients due of symptoms such as malabsorption, reduced appetite, and dietary restrictions. KGM has been shown to induce a feeling of fullness, help manage appetite, and support weight control. This could be particularly beneficial for IBD patients striving to maintain a healthy weight, as proper weight management is linked to better overall well-being and could positively influence the management of IBD symptoms (Shah et al., 2020). Despite its potential benefits, there are some considerations when incorporating KGM into the diet of IBD patients. Tolerance to KGM can vary, and some individuals may experience gastrointestinal discomfort when introducing new fibers. It is recommended to start with small amounts of KGM and gradually increase intake while monitoring for any adverse effects (Zhang et al., 2021b). Additionally, KGM may affect the absorption of certain medications; therefore, it is crucial for patients to consult their healthcare provider before incorporating KGM into their diet to ensure that it does not interfere with their treatment plan (Pan et al., 2024).

KGM also promotes the production of short-chain fatty acids (SCFAs), which are crucial for gut health. The increase in cecal SCFA contents observed with KGM supplementation indicates that it not only supports beneficial bacteria but also enhances the metabolic byproducts that these bacteria produce. SCFAs are known to have anti-inflammatory properties and may help in reducing the risk of CRC by maintaining gut barrier integrity and modulating immune responses (Zhang et al., 2023; Jin et al., 2025). Research indicates that a diet rich in dietary fiber, including KGM, is associated with a lower risk of developing CRC. This protective effect is thought to arise from the fermentation of dietary fibers in the gut, leading to the production of SCFAs, which may exert chemopreventive properties on colonocytes (Devaraj et al., 2019a). Clinical trials have demonstrated that adjuvant therapies incorporating KGM, such as immunochemotherapy with polysaccharide K (PSK), can lead to improved survival rates and disease-free survival in patients with curatively resected CRC (Sawai et al., 2019). These findings highlight KGM's potential not only as a dietary supplement but also as a therapeutic agent that may mitigate the adverse effects of cancer treatments and enhance overall efficacy. High-fiber diets, particularly those rich in whole grains and vegetables, are consistently associated with a lower risk of CRC (Shah et al., 2015a).

The application of KGM to hyperthyroidism treatment is a novel area of research. Hyperthyroidism, characterized by an overactive thyroid gland, leads to the excessive production of thyroid hormones, causing symptoms such as weight loss, rapid heartbeat, and increased metabolism (Behera and Ray, 2016). Hyperthyroidism can affect digestive health, leading to diarrhea or malabsorption. KGM, with its fiber and prebiotic properties, can help manage gastrointestinal symptoms by improving gut health and regularity (Kalra et al., 2021). KGM’s appetite-suppressing effects of KGM and its role in promoting satiety could potentially aid in managing weight changes associated with hyperthyroidism. By reducing appetite and caloric intake, KGM may help balance metabolic changes caused by these conditions (Gan et al., 2024). In hyperthyroidism, absorption of certain nutrients may be disrupted. KGM’s role in improving overall digestive health could support better nutrient absorption and help manage deficiencies commonly seen in thyroid disorders (Devaraj et al., 2019b). Although the use of KGM in wound dressings has already been established and has clinical support, its application in hyperthyroidism treatment is less well documented and requires further investigation. Research on KGM’s potential benefits of KGM for hyperthyroidism is ongoing, and more studies are needed to validate its effectiveness and safety in this context.

Moreover, KGM has demonstrated anti-genotoxic effects, which are vital in preventing DNA damage that can lead to cancer. Studies have shown that KGM supplementation reduces fecal β-glucuronidase activity and secondary bile acid levels, both of which are associated with increased cancer risk. This reduction in harmful metabolites may lower the precancerous risk factors in the colon (Yan et al., 2024). The prebiotic potential of KGM is further supported by its ability to stimulate the growth of beneficial gut bacteria, thereby enhancing gut microbiome diversity and activity (Deng et al., 2024). This modulation of the gut microbiome not only contributes to improved gut health but also plays a role in the prevention of CRC by potentially influencing metabolic pathways related to cancer development. KGM's mechanisms for modulating the gut microbiome include enhancing beneficial bacterial populations, increasing SCFA production, and exerting anti-genotoxic effects (Zhang et al., 2024). These actions collectively contribute to a healthier gut environment, which is crucial for reducing the risk of CRC. Future research, particularly *in vivo* studies involving human subjects, is necessary to further elucidate these mechanisms and confirm the beneficial effects of KGM on gut health and cancer prevention (Ye et al., 2021).

### 4.3 Anti-inflammatory and immune regulation

Regular consumption of KGM has been shown to significantly alleviate skin inflammation, particularly in models of atopic dermatitis. KGM, a dietary fiber derived from the konjac plant, possesses notable anti-inflammatory properties that contribute to its effectiveness in managing skin conditions (Pan et al., 2024). Specifically, pulverized konjac glucomannan (PKGM) has been demonstrated to reduce the frequency of skin inflammation and associated symptoms in murine models. In studies involving PKGM, mice exhibited a marked decrease in the IL-4/IFN-γ ratio in the colonic lamina propria, indicating an improvement in immune response and a reduction in inflammation (Tian et al., 2023). Furthermore, the administration of PKGM not only mitigated skin inflammation but also suppressed hyper-IgE production, which is often linked to allergic reactions and dermatitis (Devaraj et al., 2019b). The anti-inflammatory activity of KGM is prominently demonstrated through its effects in a mouse model of oxazolone-induced colitis (OXA), where it significantly ameliorated the symptoms associated with this condition. In the context of OXA-induced colitis, KGM administration resulted in a marked reduction in the levels of critical inflammatory cytokines, specifically interleukin-4 (IL-4) and interleukin-13 (IL-13) (Onitake et al., 2015). These cytokines are pivotal in mediating inflammatory responses, and their suppression indicates a robust anti-inflammatory effect of KGM. The study revealed that PKGM not only improved histological markers of colonic inflammation but also led to a decrease in the population of NK1.1+ T cells in the liver, which is associated with the induction of Th1-polarized immune responses. The mechanism underlying KGM's anti-inflammatory activity appears to involve cytokine regulation, as it influences the levels of pro-inflammatory cytokines such as TNF-α and IL-1β, which are crucial in inflammatory processes (Handa et al., 2015). By modulating these cytokines, KGM can effectively reduce inflammation and enhance immune responses, showcasing its potential therapeutic applications in inflammatory diseases. Moreover, the study highlights that the preventive role of KGM in OXA-induced colitis was not observed in invariant natural killer T cell-deficient mice, further emphasizing the importance of immune modulation in its mechanism of action (Ye et al., 2021; Fang et al., 2023b).

Emerging research suggests that dietary fibers such as KGM may have anti-inflammatory properties. This fiber has the potential to modulate the inflammatory response in the gut, which is a key aspect in managing irritable bowel disease (IBD). Although preliminary findings are promising, more studies are required to fully understand the extent of KGM's anti-inflammatory effects of KGM and its practical applications for reducing inflammation in individuals with IBD (Changchien et al., 2021). KGM has shown significant potential in wound care due to its unique physical and chemical properties, making it an effective component in modern wound dressings. The primary benefit of KGM-based wound dressings is their ability to form gel-like substances when hydrated, which offers several advantages in wound management. One of the key features of KGM in wound dressings is its ability to maintain a moist environment around the wound. A moist environment is critical to promote faster and more efficient wound healing. This moisture prevents the wound from drying out and forms a crust, which can impede the healing process. KGM dressings help keep the wound bed hydrated, facilitating cell migration and tissue repair, ultimately accelerating the healing process (Zhou et al., 2022). Another important benefit of KGM is its ability to absorb wound exudates. As the gel formed by KGM absorbs excess wound fluid, it helps to manage the moisture levels at the wound site, reducing the risk of maceration. Maceration, which occurs when the skin becomes soft and breaks down owing to prolonged exposure to moisture, can lead to further complications in wound healing. By effectively managing the exudate, KGM dressings contribute to a more stable and controlled healing environment (Borbolla-Jiménez et al., 2023). KGM is highly biocompatible and non-toxic, making it particularly suitable for use in medical applications, such as wound care. It does not cause irritation or allergic reactions, thereby ensuring patient comfort and safety during the healing process. The non-reactive nature of KGM allows its use in a wide range of patients, including those with sensitive skin or allergies, making it a versatile and safe option for wound management (Veerasubramanian et al., 2018). In some formulations, KGM wound dressings are combined with antimicrobial agents to prevent infections, which can be a major complication of wound care. These antimicrobial KGM dressings help reduce the risk of bacterial infections at the wound site, support optimal healing, and minimize the chances of further complications. This combination of antimicrobial properties and KGM's natural benefits enhances the efficacy of these dressings in clinical settings (Alven et al., 2022).

**Figure 3:**
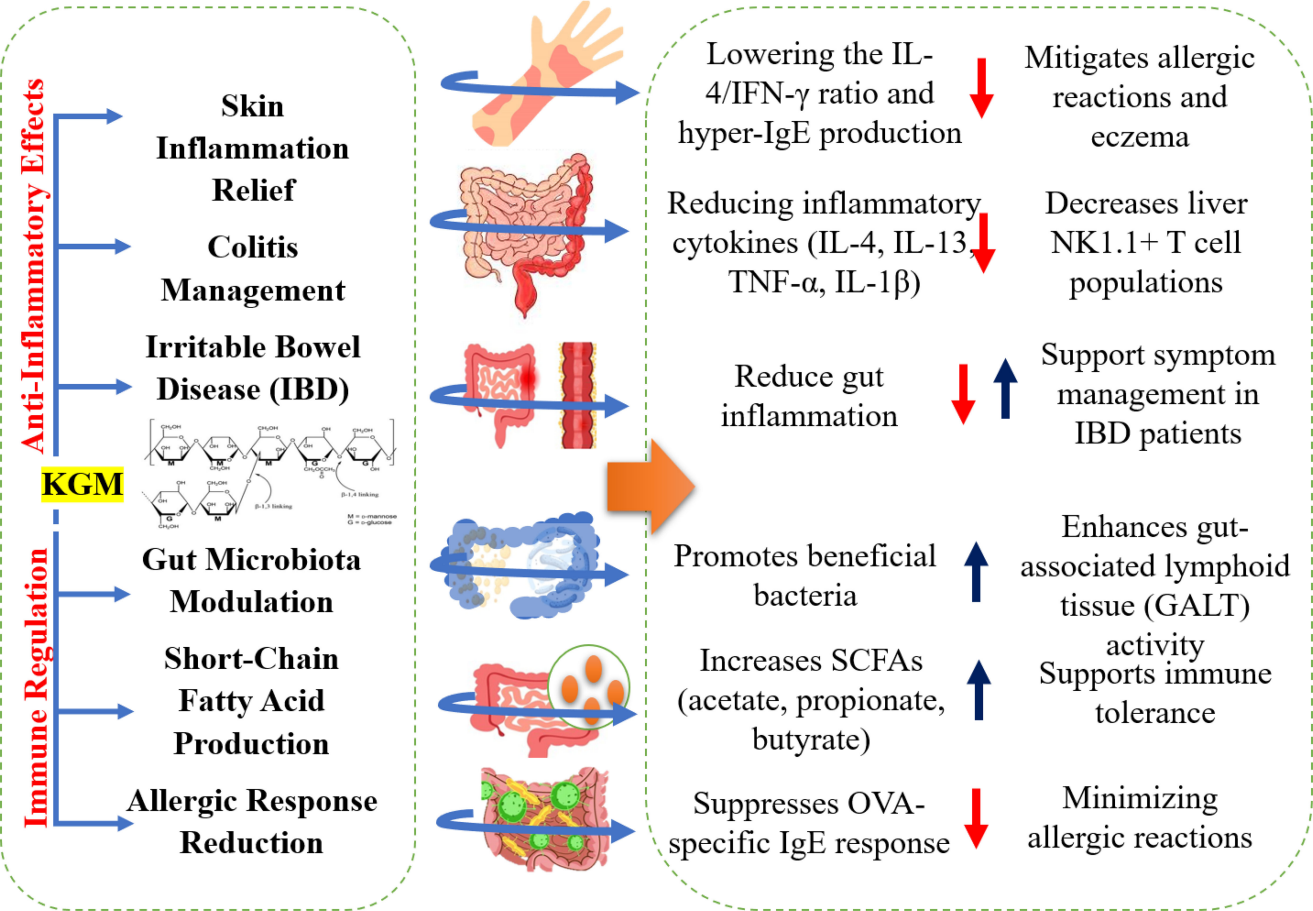
Anti-inflammatory and immune-regulating properties of konjac glucomannan (KGM).

KGM may influence the immune system primarily through its positive effects on gut health and its ability to modulate inflammation. One of the key mechanisms by which KGM supports immune function is through the modulation of the gut microbiota. Acting as a prebiotic, KGM promotes the growth of beneficial gut bacteria, such as Bifidobacteria and Lactobacilli, which are crucial for maintaining a balanced gut microbiome (Ye et al., 2021). Because a significant portion of the immune system is located in the gut-associated lymphoid tissue (GALT), the health of the gut microbiome directly affects immune responses. By promoting a healthy microbiome, KGM plays a role in strengthening immune defenses (Devaraj et al., 2019b). Another important way to support the immune system is through the production of SCFAs during fermentation by gut bacteria. SCFAs, including acetate, propionate, and butyrate, are essential for maintaining gut integrity and reducing inflammation, which can influence systemic immune responses (Zhang et al., 2022b). By promoting immune tolerance, KGM can help prevent excessive or inappropriate immune reactions, potentially reducing the risk of autoimmune disorders (Elshaghabee and Rokana, 2021). Additionally, a healthy gut microbiome is key to the development and activity of immune cells, such as T-cells and macrophages, which are central to immune defense mechanisms (Pan et al., 2024). Specifically, PKGM supplementation has been reported to suppress the OVA-specific IgE response, which is a critical marker of allergic reactions, without adversely affecting other immune responses such as IgG1 and IgG2a production (Suzuki et al., 2010). In addition to its effects on allergic rhinitis, KGM has demonstrated efficacy in preventing skin inflammation associated with atopic dermatitis (Guerreiro et al., 2023). Studies have shown that PKGM can ameliorate eczema and hyper-IgE production in mouse models, suggesting its potential as a dietary intervention for managing atopic diseases (Mao et al., 2022). However, it is important to note that the effects of KGM on the immune system vary among individuals. Those with immune-related disorders should consult healthcare professionals before incorporating KGM into their diet to ensure their safety and efficacy in specific health conditions (Srividya et al., 2024). Overall, KGM’s ability to support gut health and produce beneficial metabolites, such as SCFAs, positions it as a potential modulator of immune function (**Figure 3**).

## 5 Emerging cutting-edge research

KGM is emerging as a significant player in cutting-edge research within the fields of drug delivery and biological materials. KGM, derived from the corms of *A. konjac*, exhibits unique properties that make it an attractive candidate for various applications, particularly in the pharmaceutical sector due to its biocompatibility and biodegradability (Kulkarni et al., 2024; Zhuang et al., 2024). Recent studies have focused on the development of KGM-based microcapsules, which serve as innovative carriers for drug delivery. These microcapsules are prepared using a piercing method, where KGM forms the main membrane, encapsulating active ingredients such as N-Methyl-2-pyrrolidone (NMP) (Spirk, 2018; Khodadadi Yazdi et al., 2020). The preparation process involves blending KGM with Xanthan gum to create a gel that enhances the viscosity and tenacity of the microcapsules, thereby improving their drug delivery characteristics (Lin et al., 2024). The resulting konjac micro-balls (KMBs) demonstrate sustained-release properties, with delivery times exceeding 24 hours, indicating their potential for long-term therapeutic applications (Zhang et al., 2014). Key performance metrics such as embedding rate and encapsulation yield are critical in assessing the effectiveness of these KGM-based microcapsules. The embedding rate reflects the efficiency of drug loading, while encapsulation yield quantifies the success of the microcapsule formation process (Zhang et al., 2024). These metrics are essential for optimizing drug delivery systems, ensuring that therapeutic agents are effectively delivered to target sites within the body. Moreover, the preparation of KGM gel through alkali catalysis has opened new avenues for research in material science. The gel's properties, influenced by factors such as concentration, pH, and temperature, contribute to its potential applications in drug delivery systems (Sun et al., 2023). The molecular structure of KGM gel, characterized by advanced techniques like Fourier transform infrared spectroscopy and wide-angle X-ray diffraction, reveals a more regular arrangement compared to pure KGM, enhancing its functional capabilities in biological applications (Yang et al., 2017). The integration of KGM in drug delivery and biological materials research highlights its versatility and potential. The ongoing exploration of KGM-based systems promises to yield innovative solutions for effective drug delivery, addressing critical challenges in the pharmaceutical industry and paving the way for future advancements in health care (Zhou et al., 2022; Zhuang et al., 2024).

Furthermore, KGM serves as the primary membrane in the formation of microcapsules, which are designed to enhance drug delivery through their ability to control release and target specific sites within the body (Zhang et al., 2024). The encapsulation yield, which measures the effectiveness of KGM in encapsulating therapeutic agents, is a critical factor that underscores its potential in targeted delivery systems (Kapoor et al., 2024; Madawi et al., 2024). The structural integrity of KGM is significantly influenced by its hydrogen bonding network, particularly at the O (Xia et al., 2024) and O (Islam et al., 2023) positions on the KGM ring. These key linking points contribute to the stability of the hydrogen bonding network, which is essential for maintaining the functionality of KGM in drug delivery applications (Sun et al., 2023; Zheng et al., 2024a). The stability of this network is further enhanced through the process of deacetylation, which not only improves the hydrogen bonding structures but also increases the overall performance of KGM in drug delivery systems (Sun et al., 2023; Ye et al., 2021). Moreover, the gelation performance of KGM is crucial for its application in drug delivery. The ability of KGM to form gels allows it to encapsulate and release drugs in a controlled manner, thereby improving the therapeutic efficacy of the delivered agents (Lafarge and Cayot, 2018; Kapoor et al., 2024). The drug loading capacity of KGM-based microcapsules demonstrates how its unique structure can effectively accommodate and deliver therapeutic agents, ensuring that the drugs are released at the desired rate and location (Shen et al., 2024). The embedding rate of the microcapsules, which reflects the efficiency of KGM's structure in encapsulating the core substance, is another important aspect that enhances targeted drug delivery (Cui et al., 2021). The combination of these structural features—hydrogen bonding stability, gelation properties, and effective encapsulation—positions KGM as a promising candidate for developing advanced drug delivery systems (**Table 4**).

**Table 4:** Pharmaceutical applications and drug delivery potential of konjac glucomannan (KGM).

Aspect	Findings	Citations
Biocompatibility and Biodegradability	KGM's natural origin and compatibility with biological systems make it ideal for pharmaceutical applications, including drug delivery systems.	(Kulkarni et al., 2024; Zhuang et al., 2024)
Microcapsule Development	KGM-based microcapsules prepared using piercing methods exhibit sustained-release properties, making them suitable for long-term therapeutic applications.	(Spirk, 2018; Khodadadi Yazdi et al., 2020)
Sustained-Release Capabilities	KMBs can deliver drugs over 24 hours, showcasing potential for extended-release drug formulations.	(Zhang et al., 2014)
Encapsulation Yield and Embedding Rate	Metrics like encapsulation yield and embedding rate are critical for optimizing drug delivery systems, ensuring efficient and targeted delivery.	(Kapoor et al., 2024; Zhang et al., 2024)
Gelation Properties	Gelation performance of KGM enables encapsulation and controlled drug release, improving therapeutic efficacy in drug delivery applications.	(Lafarge and Cayot, 2018; Kapoor et al., 2024)
Hydrogen Bonding Stability	Stability at O (Xia et al., 2024) and O (Islam et al., 2023) positions contributes to KGM's structural integrity, crucial for maintaining functionality in drug delivery systems.	(Sun et al., 2023; Zheng et al., 2024a)
Deacetylation Benefits	Deacetylation improves KGM’s hydrogen bonding network, enhancing its stability and performance in drug delivery applications.	(Ye et al., 2021; Sun et al., 2023)
Structural Characterization	Advanced techniques such as FTIR and X-ray diffraction reveal a more regular molecular structure in KGM gels, enhancing biological application potential.	(Sun et al., 2023; Yang et al., 2017)
Integration with Xanthan Gum	Blending KGM with xanthan gum enhances gel viscosity and tenacity, improving the drug encapsulation and release performance of the microcapsules.	(Lin et al., 2024)
Drug Loading Capacity	KGM-based systems demonstrate effective drug loading and targeted delivery, ensuring controlled release at specific sites within the body.	(Cui et al., 2021; Shen et al., 2024)
Material Science Applications	KGM gel preparation via alkali catalysis opens avenues for broader applications in biological materials and drug delivery systems.	(Sun et al., 2023)

## 6 Multi-omics analysis and high-throughput screening approaches

Multi-omics analysis and HTS are pivotal methodologies that enhance the functional evaluation of KGM, a polysaccharide known for its health benefits and applications in food and pharmaceuticals. Multi-omics analysis integrates data from various biological layers, including genomics, proteomics, and metabolomics, to provide a comprehensive understanding of KGM's biological functions and interactions within biological systems (Wang and Li, 2021). This integrative approach allows researchers to identify the molecular mechanisms through which KGM exerts its health benefits, such as its antioxidant properties and cellular protection capabilities (Zhang et al., 2021a). On the other hand, HTS facilitates the rapid assessment of KGM's biological activity by enabling the testing of numerous compounds and their effects on specific biological pathways. This method is crucial in drug discovery and functional evaluation, as it allows for the identification of active compounds that can enhance the extraction and purification processes of KGM (Zhang et al., 2021b; Shen et al., 2024). By employing HTS, researchers can efficiently screen various extraction methods and purification techniques, optimizing conditions to isolate pure KGM or its derivatives for further study (Franková and Fry, 2015; Qi and Chen, 2024). The functional evaluation of KGM is further supported by the assessment of its antioxidant activity, which can be measured through various assays that evaluate its ability to scavenge free radicals (Zhu, 2018). This evaluation is essential for understanding how KGM can protect cells from oxidative stress, a significant factor in many diseases. Additionally, the purification techniques employed to isolate KGM are critical, as they ensure that the final product retains its functional properties, including its viscosity and health benefits (Widjanarko et al., 2024). Moreover, enzymatic hydrolysis and γ-irradiation are techniques that can modify KGM to enhance its functional properties. Enzymatic hydrolysis breaks down KGM into oligo-glucomannan, which may exhibit improved bioactivity (Zhu, 2018; Zheng et al., 2024a). Similarly, γ-irradiation can induce structural changes that enhance the extraction efficiency and functional characteristics of KGM (Zheng et al., 2024b).

However, both techniques come with their own set of advantages and disadvantages. The advantages of multi-omics include comprehensive data integration and improved understanding of biological processes, which can lead to enhanced identification of therapeutic targets (Chen et al., 2021; Li et al., 2024). Conversely, the disadvantages of HTS may include high costs, the potential for false positives, and the necessity for extensive validation of results (Misra et al., 2019). These challenges can complicate the interpretation of data and may require additional resources to ensure reliability. The integration of multi-omics and HTS in KGM research holds significant potential for future studies. By combining the strengths of both approaches, researchers can gain deeper insights into KGM's properties and interactions, ultimately leading to improved applications in food preservation and drug delivery systems. For instance, understanding the gel formation mechanism of KGM, which involves molecular interactions such as hydrogen bonding and hydrophilic group interactions, can be enhanced through multi-omics data (Song et al., 2024). Additionally, chemical modifications of KGM can be systematically evaluated using HTS to optimize its properties for specific applications (Tsurumaki et al., 2015). The synergistic use of multi-omics analysis and HTS can significantly advance the functional evaluation of KGM, paving the way for innovative applications and improved product formulations in both the food and pharmaceutical industries (**Table 5**).

**Table 5:** Advanced analytical techniques and functional insights of konjac glucomannan (KGM).

Aspect	Findings	Citations
Multi-omics Analysis	Integrates genomics, proteomics, and metabolomics data to elucidate KGM's molecular mechanisms, including antioxidant properties and cellular protection capabilities.	(Wang and Li, 2021; Zhang et al., 2021b)
High-Throughput Screening (HTS)	Facilitates rapid assessment of KGM's biological activity, enabling efficient testing of extraction and purification techniques for isolating pure KGM or its derivatives.	(Zhang et al., 2021b; Shen et al., 2024)
Antioxidant Activity	KGM's ability to scavenge free radicals and protect cells from oxidative stress is essential for understanding its health benefits.	(Zhu, 2018)
Purification Techniques	Critical for preserving KGM's functional properties, including viscosity and bioactivity, during extraction and processing.	(Widjanarko et al., 2024)
Enzymatic Hydrolysis	Converts KGM into oligo-glucomannan, enhancing bioactivity and functional properties.	(Zhu, 2018; Zheng et al., 2024a)
γ-Irradiation	Induces structural changes in KGM, improving extraction efficiency and functional characteristics.	(Zheng et al., 2024a)
Advantages of Multi-omics	Comprehensive data integration improves understanding of biological processes and facilitates the identification of therapeutic targets.	(Chen et al., 2021; Li et al., 2024)
Disadvantages of HTS	High costs, potential false positives, and the need for extensive result validation pose challenges in HTS applications.	(Misra et al., 2019)
Synergistic Integration	Combining multi-omics and HTS enhances understanding of KGM properties and molecular interactions, advancing applications in food preservation and drug delivery.	(Tsurumaki et al., 2015; Song et al., 2024)
Gel Formation Mechanism	Multi-omics data improves understanding of KGM gelation through molecular interactions such as hydrogen bonding and hydrophilic group interactions.	(Song et al., 2024)
Chemical Modifications	HTS enables systematic evaluation of KGM modifications to optimize properties for specific applications.	(Tsurumaki et al., 2015)

## 7 Limitation, challenges and future directions

The quality of KGM products is influenced by several critical factors that play a significant role in their formulation and performance. Understanding these factors is essential for optimizing the efficacy of KGM-based applications, particularly in drug delivery systems. One of the primary factors is the concentration of KGM itself. The concentration directly affects the release degree of encapsulated drugs, such as cap, which is crucial for ensuring that the active ingredients are delivered effectively. Optimal conditions have been identified, with a concentration of 2.5% (w/v) KGM yielding a predicted release degree of 92.258% for cap (Chao et al., 2012). This highlights the importance of selecting the right concentration to achieve desired release profiles. Another significant factor is the embedding rate, which determines how efficiently active ingredients are incorporated into the KGM matrix. A higher embedding rate typically correlates with better delivery of the active compounds, thereby enhancing the overall quality of the KGM product (Sun et al., 2023). Additionally, the drug loading capacity is a direct measure of how well the KGM microcapsules can incorporate and deliver these active compounds, further influencing product quality (Shen et al., 2024). The encapsulation yield is also a critical parameter, reflecting the effectiveness of the microencapsulation process. A higher encapsulation yield indicates a more successful process, which is vital for the functionality of KGM products (Zhang et al., 2024). Furthermore, the physical properties of the microcapsules, such as particle size and distribution, are essential for understanding their performance. These characteristics can significantly impact the release behavior and stability of the KGM products (Halahlah et al., 2023). The preparation parameters, including mixing time, laying-time, and drying temperature, are crucial for ensuring the quality of KGM membranes. For instance, the duration of mixing affects the uniformity of the membrane, while the laying-time is important for proper formation (Yuan et al., 2017). The drying temperature, specifically at 65°C, has been shown to influence the final quality and release characteristics of the product (Ni et al., 2021). Lastly, the morphology of the KGM products provides insights into their structural characteristics, which are critical for assessing quality. Analyzing the morphology can help identify any potential issues in the formulation process that may affect performance (Sun et al., 2023; Kapoor et al., 2024). The safety and efficacy of KGM products are significantly influenced by the dosage forms and dosages used in clinical studies. A notable example is the specific dosage of 3.9 g of KGM administered daily, which was shown to effectively lower TC levels by 10% in a controlled study involving 63 healthy men (Behera and Ray, 2016; Jian et al., 2024). This dosage not only demonstrated a significant reduction in TC but also led to a 7.2% decrease in LDL-C and a 23% reduction in triglycerides, indicating a robust lipid-lowering effect (Nie and Luo, 2021; Cheung et al., 2023). This variability in dosage highlights the importance of tailoring KGM products to individual needs and health conditions to optimize safety and efficacy. In addition to dosage, the physiochemical properties of KGM can also be altered through processes such as γ-irradiation. Studies have shown that different doses of γ-irradiation (5, 20, 50, and 100 kGy) can affect the weight-average molecular weight (M_w) and apparent viscosity of KGM, which are critical factors influencing its functional properties and, consequently, its safety and efficacy (Xu et al., 2007; Jian et al., 2016).

The extraction and purification of KGM on a large scale presents significant sustainability challenges that must be addressed to minimize environmental impact. One of the primary difficulties lies in maintaining ecological balance during these processes, which often leads to adverse effects on local ecosystems (Behera and Ray, 2017; Pan et al., 2024). Resource efficiency is a crucial aspect of sustainable KGM production. The extraction and purification processes must be optimized to minimize waste and maximize the use of available resources. This involves not only improving the efficiency of the extraction methods but also considering the trade-offs between different resource efficiency objectives, such as reducing environmental impacts while ensuring cost-effectiveness (He et al., 2024). The complexity of these trade-offs highlights the need for a comprehensive approach to resource management in KGM production. Technological innovations present a promising avenue for addressing these sustainability challenges. Advancements in extraction techniques could lead to more sustainable practices that reduce environmental impacts and enhance resource efficiency (Mathura et al., 2024). However, the adoption of such technologies must be coupled with strict regulatory compliance to ensure that environmental standards are met throughout the production process. Additionally, the impact of climate change on the availability and quality of raw materials for KGM extraction cannot be overlooked. Fluctuations in climate conditions can affect the growth of konjac plants, thereby influencing the sustainability of KGM supply chains (Srzednicki and Borompichaichartkul, 2020). This underscores the importance of supply chain coordination among producers, suppliers, and manufacturers to enhance the overall sustainability of KGM production. Addressing the sustainability challenges associated with large-scale KGM extraction and purification requires a multifaceted approach that includes improved waste management, resource efficiency, technological innovations, regulatory compliance, and effective supply chain coordination. By tackling these limitations, the industry can move towards a more sustainable future for KGM production.

To enhance the future outlook for research on KGM, strategic recommendations can be made focusing on improving extraction efficiency and developing more effective functional food and drug delivery systems. Firstly, leveraging biotechnological methods to enhance the extraction efficiency of KGM from konjac tubers is crucial. Current extraction processes can be optimized through innovative biotechnological approaches, which may lead to higher yields and better quality of KGM. This aligns with the need for improved extraction techniques that can support the growing demand for KGM in various applications. Secondly, the development of KGM-based functional food and drug delivery systems should be prioritized. Research indicates that KGM can be effectively utilized in creating microcapsules that enhance the delivery of active ingredients, such as vitamins and drugs. The preparation of KGM-based microcapsules has shown promising results in terms of morphology, particle size distribution, and encapsulation efficiency, which are critical for ensuring the stability and bioavailability of the encapsulated substances. Moreover, optimizing the release properties of these systems is essential. Studies have identified key factors affecting the release degree of encapsulated compounds, such as concentration of KGM, mixing time, and drying conditions. For instance, optimal conditions for the release of a model drug were determined to be 2.5% (w/v) KGM mixed with specific parameters, achieving a predicted release degree of 92.258%. Future research should continue to explore these parameters using response surface analysis to refine the formulation processes further. Additionally, the synergistic effects of KGM with other polysaccharides, such as xanthan gum, should be investigated. This combination has demonstrated potential in creating sustained release systems, which can significantly improve the efficacy of drug delivery. Lastly, the development of innovative functional food products incorporating KGM can address health concerns such as weight management and cholesterol control. By focusing on these health benefits, researchers can create products that not only meet consumer demand but also contribute positively to public health. In conclusion, by enhancing extraction methods, optimizing drug delivery systems, and developing functional foods, future research can significantly advance the applications of KGM, making it a valuable component in health and nutrition sectors (**Table 6**).

**Table 6:** Key factors and innovations in konjac glucomannan (KGM) applications.

Aspect	Key Factors	Findings/Recommendations	Citations
KGM Concentration	Drug Release Degree	Optimal concentration: 2.5% (w/v) KGM achieves 92.258% drug release.	(Chao et al., 2012)
	Embedding Rate	Higher embedding rates improve delivery efficiency of active ingredients.	(Sun et al., 2023; Shen et al., 2024)
	Drug Loading Capacity	Higher drug loading enhances the incorporation and release of active compounds.	(Sun et al., 2023)
	Encapsulation Yield	High yield indicates successful microencapsulation and product functionality.	(Zhang et al., 2024)
Physical Properties	Particle Size & Distribution	Influences release behavior and stability, requiring precise formulation.	(Halahlah et al., 2023)
Preparation Parameters	Mixing Time, Laying-Time, Drying Temperature	Uniformity of KGM membranes and product quality depend on optimized mixing, laying-time, and drying (e.g., 65°C).	(Yuan et al., 2017; Ni et al., 2021)
Morphology Analysis	Structural Quality	Morphology analysis helps identify issues in structural quality and formulation.	(Sun et al., 2023; Kapoor et al., 2024)
Dosage and Clinical Efficacy	Specific Dosage	A daily dose of 3.9 g KGM reduces cholesterol levels (10% TC, 7.2% LDL-C, 23% triglycerides).	(Behera and Ray, 2016; Cheung et al., 2023)
	Physicochemical Modifications	γ-Irradiation modifies molecular weight and viscosity, affecting KGM's functional properties.	(Xu et al., 2007; Jian et al., 2016)
Sustainability Challenges	Environmental Impact	Address ecological challenges in extraction and purification processes.	(Behera and Ray, 2017; Pan et al., 2024)
	Resource Efficiency	Optimize extraction methods and minimize waste for cost-effective production.	(He et al., 2024)
	Climate Change Impact	Fluctuating climate affects raw material supply, requiring robust supply chain management.	(Mekkerdchoo et al., 2016)
Technological Innovations	Extraction Techniques	Advanced methods improve sustainability and reduce environmental impact.	(Mathura et al., 2024)
	Regulatory Compliance	Adhere to environmental standards to ensure sustainable production practices.	(He et al., 2024)
Extraction Efficiency	Biotechnological Approaches	Enhanced extraction efficiency and yield through biotechnological innovations.	(He et al., 2024)
Drug Delivery Systems	Encapsulation Techniques	Improve encapsulation efficiency for enhanced stability and bioavailability.	(Sun et al., 2023)
	Synergistic Effects with Other Polysaccharides	Combining KGM with xanthan gum shows potential for sustained-release drug systems.	(Tsurumaki et al., 2015)
Functional Food Innovations	Weight Management and Cholesterol Control	Develop KGM-based functional food products to address growing consumer demand for health benefits.	(Behera and Ray, 2017; Cheung et al., 2023)
Release Optimization	Formulation Refinements	Optimize key factors like KGM concentration, mixing, and drying for controlled release systems.	(Chao et al., 2012)

## 8 Conclusions

*A. konjac*, particularly its bioactive component KGM, stands as a promising dietary fiber with extensive health benefits and industrial applications. Its unique physicochemical properties, such as high viscosity and gel-forming ability, enable its use in various health and nutritional applications, including blood glucose regulation, weight management, lipid metabolism improvement, and gastrointestinal health. KGM’s prebiotic and anti-inflammatory properties further enhance its therapeutic potential, with emerging applications in wound care, hyperthyroidism management, and CRC prevention. Despite its established benefits, the continued growth of KGM-based products requires rigorous quality control measures to ensure purity, molecular consistency, and safety. Addressing potential side effects, such as gastrointestinal discomfort and allergenicity, is essential for consumer safety. Future research focusing on bioavailability, targeted delivery systems, and novel formulations can significantly enhance KGM’s therapeutic efficacy. Innovations in sustainable cultivation and ethical sourcing practices will ensure the environmental and economic viability of KGM production. Expanding applications beyond current uses, particularly in pharmaceuticals, cosmetics, and medical devices, presents an exciting avenue for KGM research. Detailed mechanistic studies on KGM’s interaction with gut microbiota and metabolic pathways will deepen understanding of its health impacts and foster the development of targeted interventions. Population-based clinical trials and epidemiological studies are critical to establishing evidence-based guidelines for KGM consumption, ensuring its long-term safety and efficacy. In conclusion, KGM represents a versatile and beneficial component in functional foods, nutraceuticals, and therapeutic applications. With advancements in research, innovation, and sustainability, KGM holds immense potential to contribute significantly to global health and wellness, supporting diverse populations while promoting sustainable and ethical practices in its production and use.
